# Identification of constraints influencing the bacterial genomes evolution in the PVC super-phylum

**DOI:** 10.1186/s12862-017-0921-3

**Published:** 2017-03-09

**Authors:** Sandrine Pinos, Pierre Pontarotti, Didier Raoult, Vicky Merhej

**Affiliations:** 1 Aix Marseille Université, CNRS, Centrale Marseille, I2M UMR 7373, Evolution Biologique et Modélisation, 3 place Victor Hugo, Marseille, 13331 France; 2 Aix Marseille Univ, CNRS, IRD, INSERM, AP-HM URMITE, IHU -Méditerranée Infection, 19-21 Boulevard Jean Moulin, Marseille, 13005 France

**Keywords:** Horizontal transfer, Bacteria, Environments, Lifestyle, Genomes, Functions

## Abstract

**Background:**

Horizontal transfer plays an important role in the evolution of bacterial genomes, yet it obeys several constraints, including the ecological opportunity to meet other organisms, the presence of transfer systems, and the fitness of the transferred genes. Bacteria from the *Planctomyctetes, Verrumicrobia, Chlamydiae* (PVC) super-phylum have a compartmentalized cell plan delimited by an intracytoplasmic membrane that might constitute an additional constraint with particular impact on bacterial evolution. In this investigation, we studied the evolution of 33 genomes from PVC species and focused on the rate and the nature of horizontally transferred sequences in relation to their habitat and their cell plan.

**Results:**

Using a comparative phylogenomic approach, we showed that habitat influences the evolution of the bacterial genome’s content and the flux of horizontal transfer of DNA (HT). Thus bacteria from soil, from insects and ubiquitous bacteria presented the highest average of horizontal transfer compared to bacteria living in water, extracellular bacteria in vertebrates, bacteria from amoeba and intracellular bacteria in vertebrates (with a mean of 379 versus 110 events per species, respectively and 7.6% of each genomes due to HT against 4.8%). The partners of these transfers were mainly bacterial organisms (94.9%); they allowed us to differentiate environmental bacteria, which exchanged more with *Proteobacteria*, and bacteria from vertebrates, which exchanged more with *Firmicutes*. The functional analysis of the horizontal transfers revealed a convergent evolution, with an over-representation of genes encoding for membrane biogenesis and lipid metabolism, among compartmentalized bacteria in the different habitats.

**Conclusions:**

The presence of an intracytoplasmic membrane in PVC species seems to affect the genome’s evolution through the selection of transferred DNA, according to their encoded functions.

**Electronic supplementary material:**

The online version of this article (doi:10.1186/s12862-017-0921-3) contains supplementary material, which is available to authorized users.

## Background

The extensive amount of genomic data acquired over the last 20 years has provided insights into the evolutionary processes that drive bacterial evolution. The horizontal transfer of DNA (HT) appears to be major driving force of innovation [1, 2] as it provides additional functions, allowing adaptation to specific conditions and environmental changes. The HT process in bacteria depends on several conditions [3]: i. the possibility of exchanges, meaning the presence of different microorganisms in a single place; ii. the possibility of foreign sequences to enter into recipient bacteria, mediated by conjugation, transformation or transduction; iii. the ability to integrate into the recipient genome; iv. the genes expressed and the genes used v. those conserved, in relation to the benefits for recipient bacteria. This process could be regulated by intrinsic and extrinsic constraints. Two extrinsic constraints influencing the possibility of exchanges include the environment or the “ecological niches” and the lifestyle, which together constitute the habitat of bacteria [4–6]. Thus the proportions and origins of HT were more similar among bacteria from the same habitat than among bacteria from a given phylum [7]. Changing environmental conditions are also well known constraints for HT regulation; UV irradiation or starvation and other stress conditions, were shown to affect the mobility of transposons and insertion sequences [6, 8–10]. The habitat also seems to play an important role in the selection and conservation of transferred sequences encoding for specific functions that are involved in host’s colonization, and the development of pathogenesis. Indeed, many examples in the literature indicate that genes encoding for metabolic functions [11–13] and for antibiotic resistance [14, 15] and virulence [16–18] represent commonly transferred sequences. The intrinsic constraints that influence the entrance and integration of foreign DNA into a recipient genome include the exclusion surface that limits the entrance of specific sequences in some bacteria [3], the presence of CRISPR that decreases the quantity of transferred sequences insertion in recipient genomes [19, 20] and the presence of some endo-nucleases that can destroy foreign DNA [3, 21].

Many studies have been conducted to explore the impact of the different extrinsic and intrinsic constraints on horizontal transfers. However, these studies involved one or a few species, or bacteria presenting only one to two habitats or lifestyles [22–25], or undergoing relatively few intrinsic constraints [26, 27]. The study of only few characteristics may one lead to miss the cumulative or overlapping effects of the different constraints. Therefore, we used a phylogenomic approach to mine a large set of bacteria with different habitats in order to decipher the impact of different constraints on genome composition, especially regarding HT. The PVC super-phylum seems to be a good model to study, as it includes seven bacterial phyla (*Planctomycetes, Verrucomicrobiae, Chlamydiae, Lentisphaera, Poribacteria, OP3, WWE2*) [28–31] with diverse habitats, three different lifestyles (intracellular allopatric, intracellular sympatric, extracellular sympatric) and numerous environments (water, soils, water and soils, metazoa, amoeba, ubiquitous…), thus varying the external constraints. Moreover, a specific cell plan is also present in all the *Planctomycetes* [32–34], in some of *Verrucomicrobiae* [35] in one *Lentisphaera* and in one *Poribacteria* [36]. The cytoplasm of these bacteria is separated into two compartments by an intracytoplasmic membrane (ICM), the pirellulosome inside (with DNA [37]) and the paryphoplasm outside. This membrane is a lipid bilayer in contact with proteins [32, 33, 38] presenting structural similarities with proteins from eukaryotic membranes like the clathrins [39, 40]. The function of this intracytoplasmic membrane is still unknown, but we hypothesize the possible impact of this intrinsic constraint on HT. In the present investigation, we analyzed 33 PVC bacteria together with 31 phylogenetically close species (*Bacteroidetes, Chlorobi* and *Spirochaetes)* that were considered as the control group, looking for evidence for horizontal transfer. Statistical analyses of the potential partners and functions involved in HT allowed us to estimate the real impact of habitat and cell plan on the genomes evolution.

## Methods

### Bacterial set selection, definition of lifestyles, environments and cell plan

The genomes of 64 bacteria have been retrieved from two different databases [41, 42]. These bacteria belong to different phyla (Additional file 1) including four phyla of the PVC super-phylum, *Planctomycetes*, *Verrucomicrobiae, Lentisphaerae* and *Chlamydia* and the phylogenetically closest phyla, *Bacteroidetes, Chlorobi* and *Spirochaeta* (determined thanks to a reference tree [43]). We reconstructed the species tree of PVC bacteria and *Bacteroidetes-Chlorobi-Spirochaetes* on the basis of 12 markers that are common to the 64 species (Additional file 2) using Mega5 [44]. Therefore, the protein sequences of each marker were aligned with Muscle [45] and non-conserved positions were removed manually. All alignments were concatenated, leading to an alignment of 5067 sites. We used a Maximum likelihood tree (substitution model JTT) based on this concatenated alignment to reconstruct the phylogeny of species. Bootstrap support values were obtained with 150 replicates (Additional file 3). The bacteria studied have different lifestyles (intracellular or extracellular, allopatric or sympatric [46, 47]) and live in different environments (amoeba, mammals, soils, water, insects). We use the term “environment” as the main place where the bacteria are living. For example, bacteria detected in sea, freshwater or wastewater are all annotated as bacteria from ‘water’. If bacteria are present in two different environments, we indicate both of them (for example ‘water-soil’ bacteria), while bacteria living in more than 5 environments are considered ‘ubiquitous’. The lifestyle of bacteria is characterized by two factors: the intracellular and extracellular conditions, and the ability to exchange with other microorganisms (in allopatric or sympatric lifestyles, respectively [46]). Lifestyles and living environments were defined for each bacterium based on a literature search [48–53]. Cell plans of the bacteria were determined via transmission electron microscopy images already available in the literature [33–36] and microscopic observations of the bacteria realized in our laboratory [54]. Three states are determined for the cell plan: compartmentalization, non compartmentalization and unknown. The selected set of species contains 4 bacteria from amoeba, 4 from insects (1 intracellular, 3 extracellular), 3 from soils, 4 living in soils and water, 3 ubiquitous, 26 from vertebrates (18 extracellular, 8 intracellular) and 20 from water. Among these 64 bacteria, 20 present a compartmentalized cell plan, 5 have an unknown cell plan, and 39 are not compartmentalized (Additional file 1).

### Genome analysis: common genes, specific genes, ORFans

OrthoMCL [55] was used to obtain groups of orthologous proteins. Groups containing at least one representative member of each habitat were considered as the common genes, and those that contain only proteins of bacteria from the same habitat are considered as specific to the corresponding habitat. We calculated the rate and determined the function of proteins that are specific to habitat in each species using two software, COGnitor and WGA and the Interpro database [56–59]. Genes that do not belong to any orthologous group are either acquired by “specific” HT or generated de novo. Blast against NR database allowed the identification of ORFans in the genome with no identifiable homologous (i.e. genes that do not have a Blast hit with an *e*-value < 10e-4 AND a query coverage >50%). We performed a clustering of all species according to their genes contents in order to detect, in some bacteria, a tendency to share the same gene contents in relation with their habitat.

### HT detection, functions and partner identification

Generally, two main difficulties hinder the analysis of the Horizontal Transfer of DNA sequences (HT) according to habitat: the distinction between the ancestral and recent gene gains, as well as the difficulty of determining the ancestral habitat of the bacteria. In order to avoid these problems, we focused our study on recent transfers that occurred only in modern species of the super-phylum (in the leafs of the tree) and not in their ancestors (at the nodes of the tree). HT instances were identified using a comparative phylogenomic approach, phylogenetic profiling of proteins and phylogenetic analysis of gene trees in comparison with the species tree. Using the Phylopattern [60] pipeline, we identified the gene gain events in four steps: 1) based on the orthologous groups, a tree was reconstructed for each group. 2) The topologies of these trees were compared with that of the species tree in order to detect species missing in orthologous groups. 3) We obtained a pattern of presence/absence for each gene in the different species, allowing Phylopattern to reconstruct the ancestral states of genes by implementing the Sankoff parsimony algorithm [61]. Based on this reconstruction, the pattern-matching module in PhyloPattern allows us to infer, by parsimony, two types of genetic events that could have occurred during gene evolution: gains and losses. The gains could be a possible HT, de novo genes or artifacts, therefore, among gene gains detected by Phylopattern, we had to identify those due to HT. We focused on specific gene gains and performed a Blast to identify similar sequences in the NR database. If the first twenty hits of Blast result belong to species outside the super-phylum of the query, and they are orthologous as confirmed by a reciprocal best hit, the enquired gene could be considered horizontally acquired. The pattern permitting the automatic identification of HT among gain events is presented in Additional file 4. Sequences with *e*-value > 10–5, coverage < 60% or identities < 30% were not considered. The localization of these events in the genome allowed the identification of any horizontal transfers of DNA sequences. The directionality of the transfer could not always be identified, but as we were interested in the capacity of exchange of the bacteria, it did not matter if the bacterium was the donor or the recipient. If some transferred genes are side by side in genomes, and were exchanged with the same partners, we considered them to have been transferred by a single event.

We calculated quantities and proportions of proteins (proteins transferred/total proteins in proteome) and sequences (nucleotides transferred/total nucleotides in genomes) implicated in HT for each genomes and the size of the transferred sequences. We identified the function of transferred genes by using two software programs, COGnitor and WGA and the Interpro database [56–59]). These programs attribute one type of function to proteins, according to the COG to which proteins belong. We studied the possible significant differences in HT distribution among the different studied groups of bacteria concerning the HT partners and functions.

### Statistical analyses

The occurrence of HT and the frequency of specific genes were compared among the different groups of bacteria from different habitats. We tested whether the data (proportions of functions for specific genes and proportions of partners and functions for HT) follows a normal (Gaussian) distribution using the Shapiro-Wilk test and we controlled the homogeneity of the data by the Levene test [62]. These tests were followed by a comparison of variance among the different habitats, using the Kruskal test [63] or the ANOVA test, according to whether or not the data had a normal distribution. The Nemenyi [64] or Tukey [65] tests were performed to obtain a comparison of each pair of habitats. We also realized a Principal Components Analysis (PCA), focused on HT proportions in genomes, size, functions and partner of transfer, followed by a hierarchical clustering (HCPC), to identify clustering of bacteria according to their transfer partners or their functions. All analyses were realized with R software.

We then used comparative phylogenetic methods to test the impact of phylogenetic relationships between species on the acquisition of studied characters. Analysis of variance was used in intergroup comparisons of categorical variables to determine whether there is statistical significance of the repartition of species on the basis of their habitat, compared to their classification according to phylogenetic distances (Additional file 3). Therefore, the Nemenyi [64] or Tukey [65] tests were performed to obtain a comparison of each pairs of groups based on the phylogenetic relationships. These groups were determined by the phylogenetic distance separating bacteria. We compared the results of these tests with the results of tests carried out for groups based on habitats, allowing us to determine if classes defined by the phylogenetic distances present different and more reliable results than classes based on habitat (t-test). If results were similar, it could be difficult to determine whether the differences observed were related to the habitat or to the phylogenetic relationships. We also performed two correlation tests. The first test, Pagel’s correlation method [66], performed on Mesquite, is a test of the independent evolution of two binary characters (all features studied were tested thanks to a binarization of continuous values). This test compares the ratio of likelihoods of two models where the rates of change in each character are dependent or alternatively independent from phylogenetic relationships. The second test is the Spearman coefficient [67], weighted by the phylogenetic distances that studies the relationship between two variables. The detection by means of this correlation test of a significantly convergent character in bacteria from a single habitat is rather unrelated to the phylogenetic background.

## Results

### Pangenome analysis

**Fig. 1:**
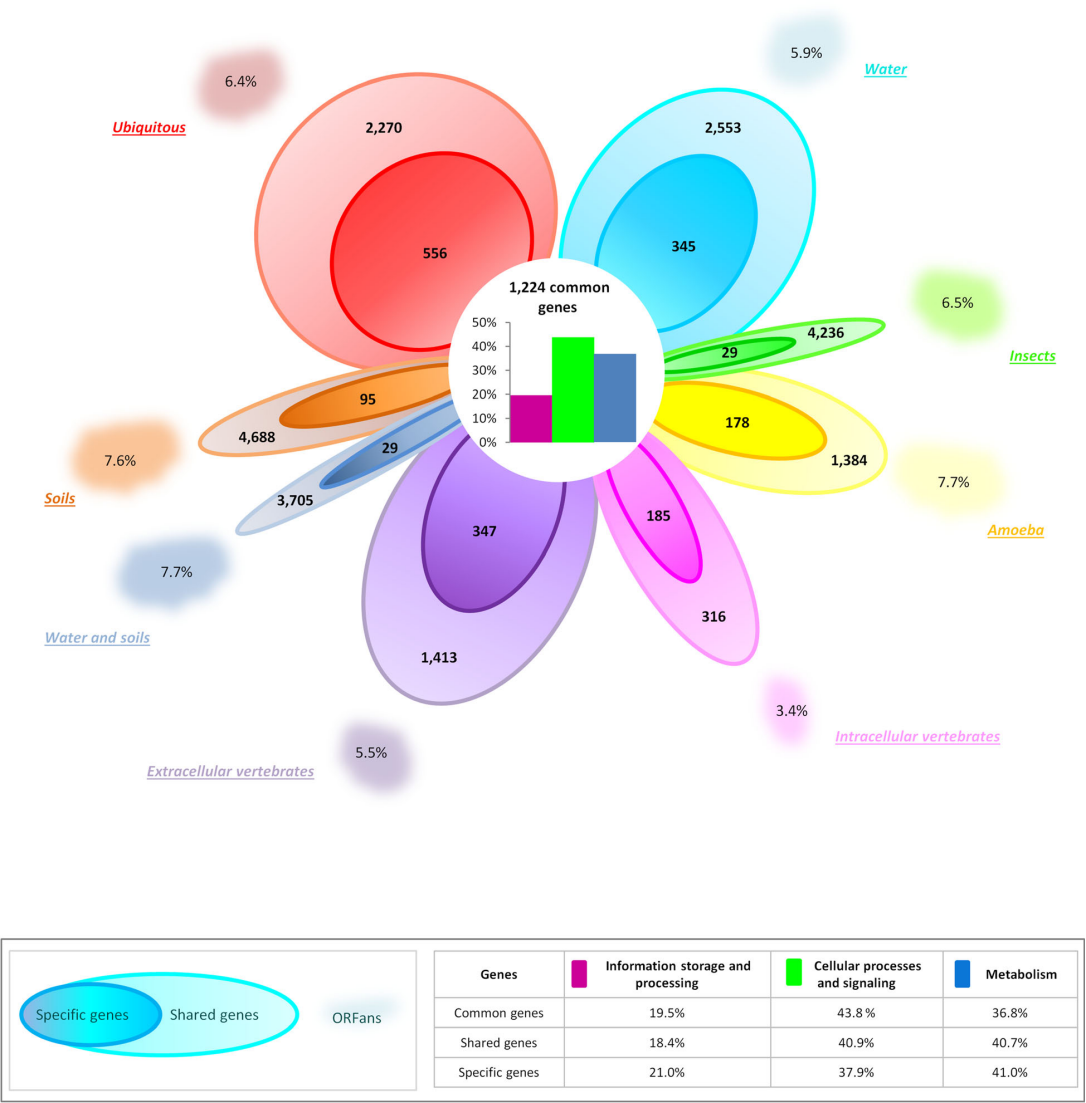
Comparison of the genome contents in the different habitats. The quantity of genes common to all habitats (*common genes*), and their functions, are indicated in the center of the flower. Genes specific to each habitat and genes shared by different habitats are represented by the different *petals*. The *clouds* represent the ORFans percentages in genomes. The *width of the petals and the clouds* is proportional to the quantity of specific genes and percentage of ORFans, respectively. The functional distribution of common, shared and specific genes is presented in the table

The genome size varied widely among the 64 bacteria studied, ranging from 0.63 Mb for *Blattabacterium* to 9.76 Mb for *Singulisphaera acidiphila* with an average of 3.83 +/− 2.2 Mb. Bacteria from soils, insects and water-soils presented the largest genome sizes, with an average of 7.07, 6.45 and 5.69 Mb, respectively, while the smallest genome sizes were found among intracellular and extracellular bacteria of vertebrates, and bacteria from amoeba, with an average of 1.14, 2.56 and 2.91 Mb, respectively. Ubiquitous bacteria and bacteria from water with an average genome size of 4.63 Mb, formed the medium-sized genomes. Likewise, the protein sets were very different among the studied bacteria, ranging from 579 proteins for *Blattabacterium* sp to 7969 for *Gemmata obscuriglobus*, with an average of 3227 +/− 2513. When using OrthoMCL, 124,175 out of 206,508 proteins that form the pangenome of the PVC group bacteria, could be assigned to 16,918 different orthologous groups (OG). Among these, 1224 OGs were common to the eight different habitats studied, and constituted the common genes. The rest of the genes were present in bacteria from two or more habitats, and thus form the “shared genome.” or they did not share sequence similarity with any other gene of the species of other habitats, and thus constituted the “specific genes” (Fig. 1). When species were clustered according to their gene contents, some bacteria sharing a same ecological niche were preferentially grouped together, forming subclusters within the clusters, determined by phylogenetic relationships or disturbing the phylogenetic unity of some groups, like the *Verrucomicrobiae, Spirochaetes and Planctomycetes* (Additional file 5). Thus the ecological niche seems to have influenced the gene content of some bacteria from soil (*p*-value = 0.027) and water (*p*-value = 0.045 for internal cluster). Bacteria from insects that showed the highest proportion of their genes shared with the other groups and the lowest quantity of specific genes (29 genes) were scattered throughout the different clusters (Additional file 5).

The genes that are common to all habitats represented 26.2% of the content of each genome on average, and varied from 20.1% for bacteria from insects to 49.5% for the intracellular vertebrates. In order to determine the functional profile of the common genes, each protein was assigned to Cluster of Orthologous Groups of proteins (COGs) functional category. We could infer a putative function to the protein sequences of 74% of the common genes; of these, 43.8% encode for cellular processes and signaling, 36.8% for metabolic functions, and 19.5% for storage and processing information (Fig. 1). Among these, four functions were significantly over-represented compared to the other functions: wall/membrane/envelop biogenesis, signal transduction mechanisms, transcription and energy production and conversion (12.4, 11.6, 9.1 and 8.5%, respectively Chi2 test: *p*-value = 9.4*10-7) (Fig. 1).

**Fig. 2:**
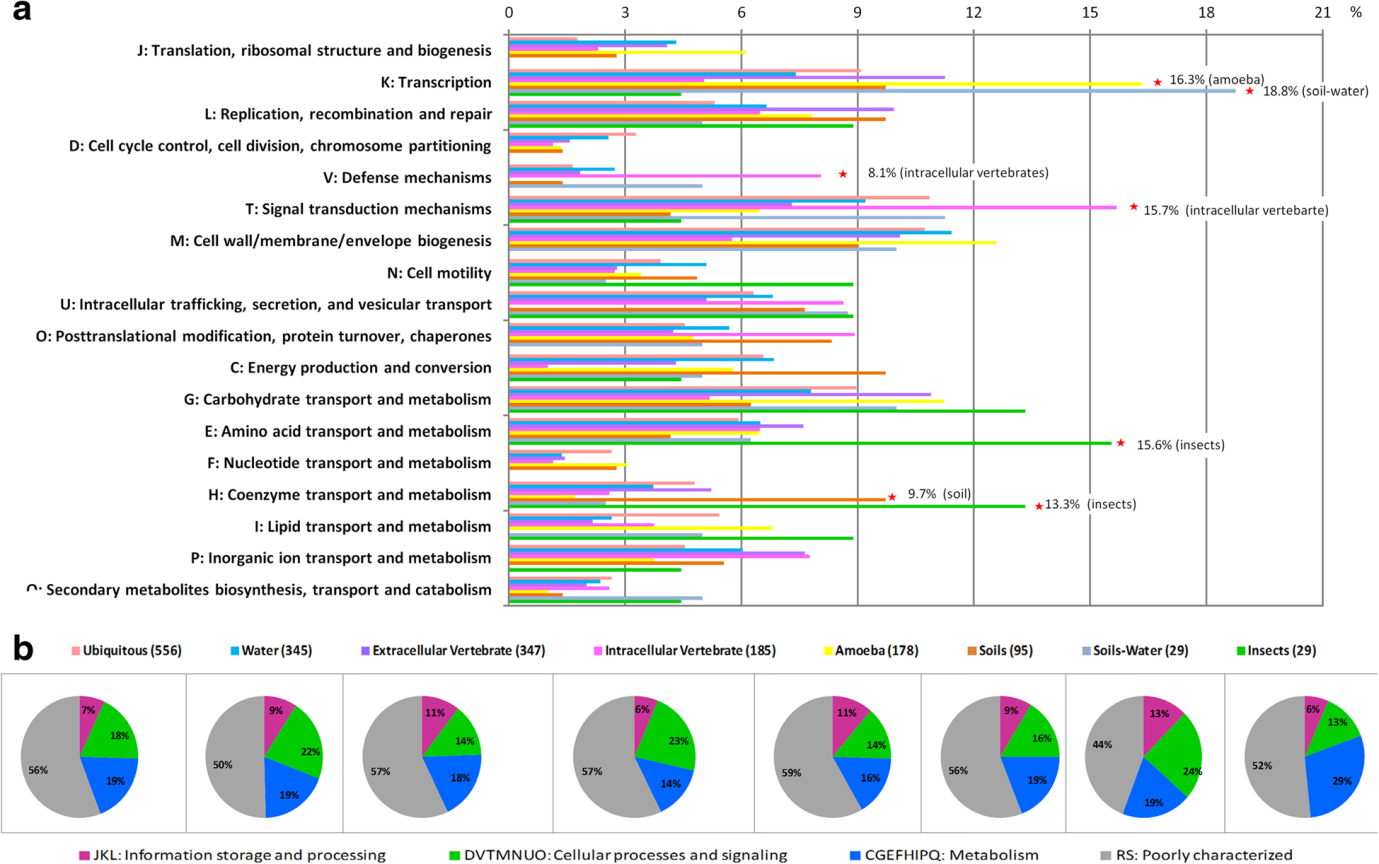
Functional distribution of the specific genes. **a** Proportions of each COG categories in the bacteria from different habitats. **b** The repartition of the four functional categories in each studied habitat. The *red stars* indicate the overrepresented functions in a habitat, compared to the other habitats, we also indicated the percent of specific genes supporting these functions

The genes shared by some habitats and the specific genes represented 35.3 and 8.1% of the content of each genome, respectively on average. The specific genes varied from 0.5% in bacteria from insects to 18.4% in the intracellular vertebrates. Of all species analyzed, *Bacteroides xylanisolvens* and *Bacteroides vulgatus* in the group of extracellular vertebrates, had the most specific genes, with a total of 801 and 780 exclusive sequences (18.2 and 19.2% of their total genomes), respectively. The functional distribution of the specific genes was significantly different from that of the common genes in all the habitats with fewer genes implicated in cell process and signaling (37.9%) (t-test for comparison between specific genes and common genes; *p*-value = 4.3*10-4) (Fig. 1). Some functions were significantly over-represented in some habitats compared to other habitats (Fig. 2), including transcription within their specific genes (16.3 and 18.8%, Kruskal-Wallis test: *p*-value = 3.9*10-6 and Correlation test : *p*-value = 4.7*10-3) in bacteria from amoeba and from soils-water; the signal transduction mechanisms and defense mechanisms in the intracellular bacteria of vertebrates (15.7%, Kruskal-Wallis test : *p*-value = 2.2*10-5 and 8.1%, 1.8*10-6, respectively; Correlation test : *p*-value = 5.5*10-2 and 3.1*10-3, respectively); the transport and metabolism of amino acid and coenzyme in bacteria from insects (15.6%, Kruskal-Wallis test: *p*-value = 1.0*10-2 and 13.3%, ANOVA test : *p*-value = 1.1*10-7, respectively), the transport and metabolism of coenzymes in bacteria from soils (9.7%, ANOVA test: *p*-value = 1.1*10-7) (Fig. 2). Although they had high proportions of specific genes, ubiquitous bacteria, bacteria from water and extracellular bacteria from vertebrates had no overrepresented functional category compared to the others (Fig. 2).

### Horizontal transfers

**Fig. 3:**
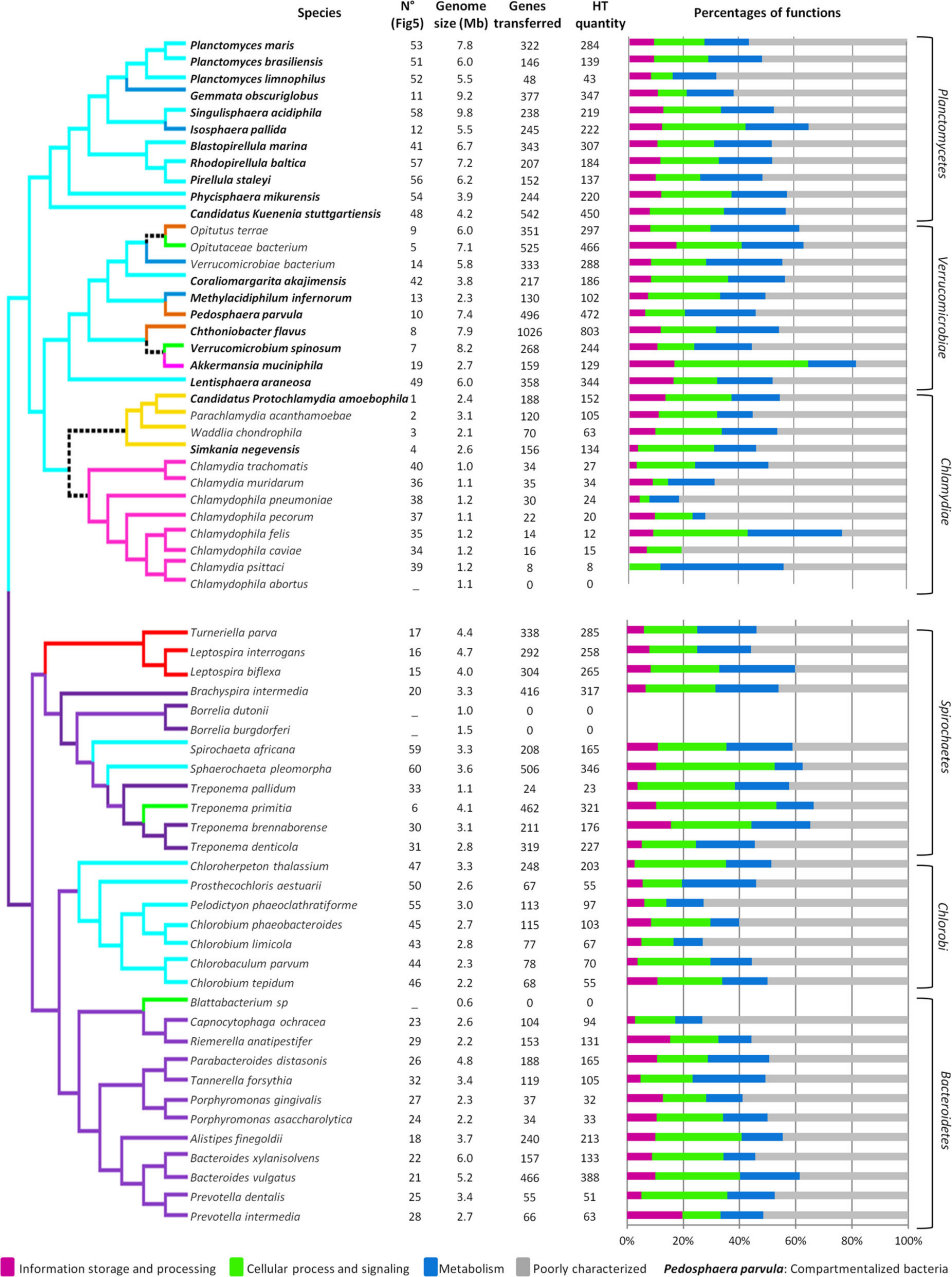
Functional description of the detected horizontally transferred genes in each studied bacteria. The habitats of species and their ancestors are indicated by the color of the *branches* (*red* : ubiquitous, *cyan* : water, *green* : insects, *yellow*: amoeba, *pink*: intracellular vertebrates, *purple*: extracellular vertebrates, *blue*: water and soils, *brown*: soils), the *black dotted branches* indicate an unknown habitat. The compartmentalized bacteria are indicated in *bold*. The first column contains the number assigned to each species in Fig. 5, the second presents the genome sizes of bacteria, the third, the quantity of genes transferred in genomes, and the fourth, the quantity of HT detected. The *bar graphic* represents the distribution of the four functional categories of COGs (Information storage and processing, Metabolism, Cellular process and signaling, Poorly characterized), which contain the 18 sub-categories studied

Phylogenetic analyses conducted to infer the evolutionary origin of all the proteins other than the common genes indicated that 12,885 proteins (6.3% of all the proteins) were acquired via horizontal transfer (Fig. 3). As transfer events are not necessarily confined to individual genes but they may concern a cluster of genes, we considered neighboring genes with the same horizontal transfer history to reflect only a single event of HT. We counted a total of 10,918 HT events among studied bacteria. The incidence with which the HT events occurred was as high as 170.6 +/− 116.4, yet this was highly variable among bacteria ranging from 0 transfers in the genome of *Blattabacterium* and *Borrelia* spp. to 803 sequences transferred in the genome of *Chthoniobacter* (Fig. 3 and Additional file 6). The count of HT events was not correlated with the genome size (Wilcoxon, *p* = 0.042) (Additional file 7). The transferred fragments length ranged from 859 bp for *C. tepidum* to 1492 pb for *S. acidophila*, with a mean length of 1124+/− 274 bp for all fragments. The size of the HT was 1022 +/− 383 bp on average, and was similar in the different habitats. Bacteria from soil, from insects and ubiquitous bacteria presented the highest average of HT (524.0, 343.7 and 269.3 transfer events per species, respectively), compared to bacteria living in water, extracellular bacteria from vertebrates, bacteria from amoeba and the intracellular bacteria of vertebrates (183.7, 126.7, 113.5 and 17.6 HT per species, respectively). The statistical comparison of the bacterial group from different habitats allowed us to define three classes, based on percentages of sequences due to transfer (Kruskal-Wallis test : *p*-value = 2.3*10-2): Bacteria of soils (8.4%) and insects (7.3%) and ubiquitous bacteria (6.6%) defined the first class. Bacteria from soil-water (5.0%), water (4.9%), amoeba (4.8%), and extracellular bacteria of vertebrates (4.6%) presented similar proportions, and formed the second class. Intracellular bacteria of vertebrates were grouped in the third class, with 1.9% of HT events (Additional files 6).

**Fig. 4:**
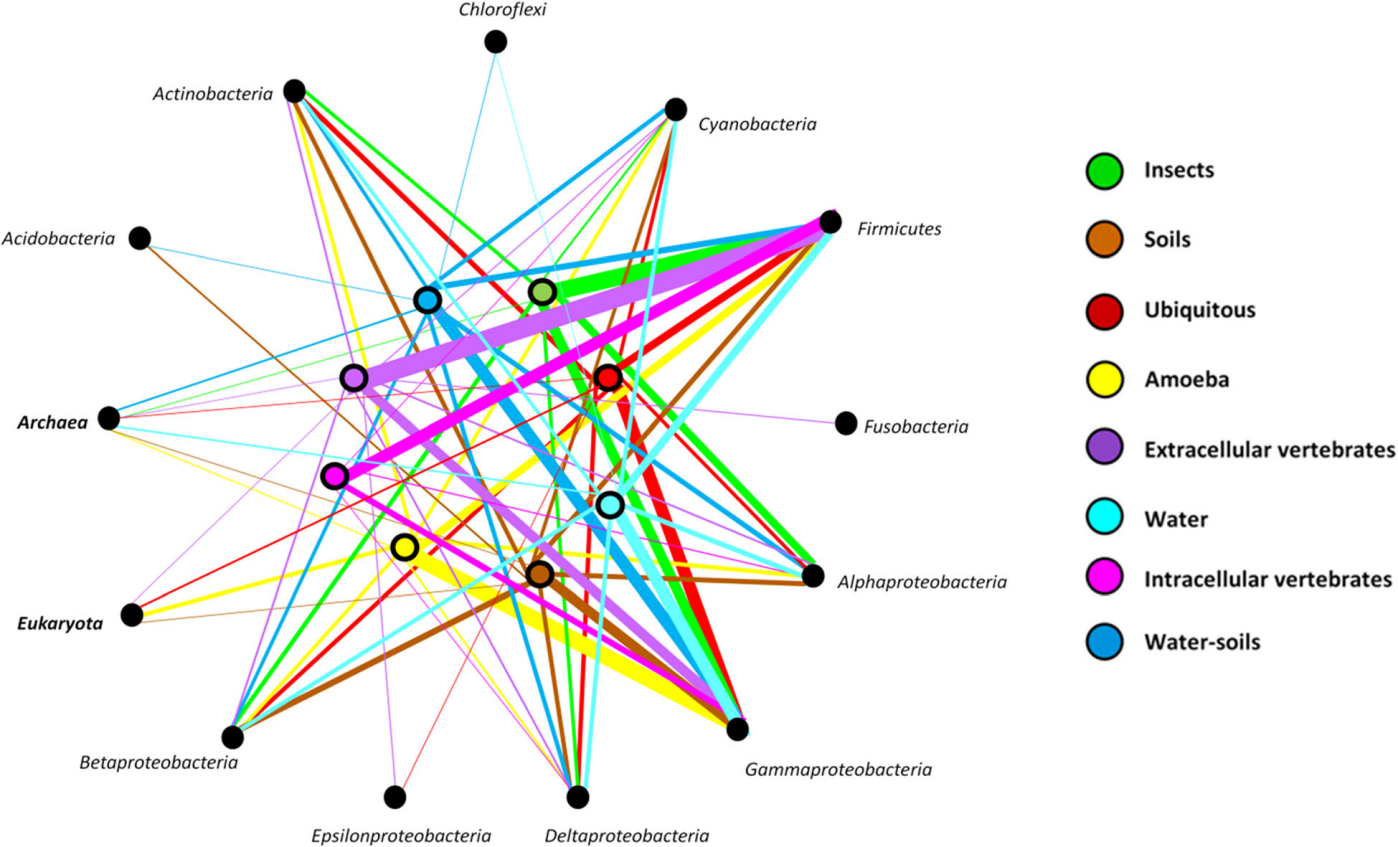
Preferences for horizontal transfer partners among habitats. The *colored point* corresponds to the bacteria from the different habitats (*red* : ubiquitous, *cyan* : water, *green* : insects, *yellow*: amoeba, *pink*: intracellular vertebrates, *purple*: extracellular vertebrates, *blue*: water and soils, *brown*: soils). The traits are colored according to the habitats of studied bacteria and their thickness is proportional to the amount of genes exchanged

**Fig. 5:**
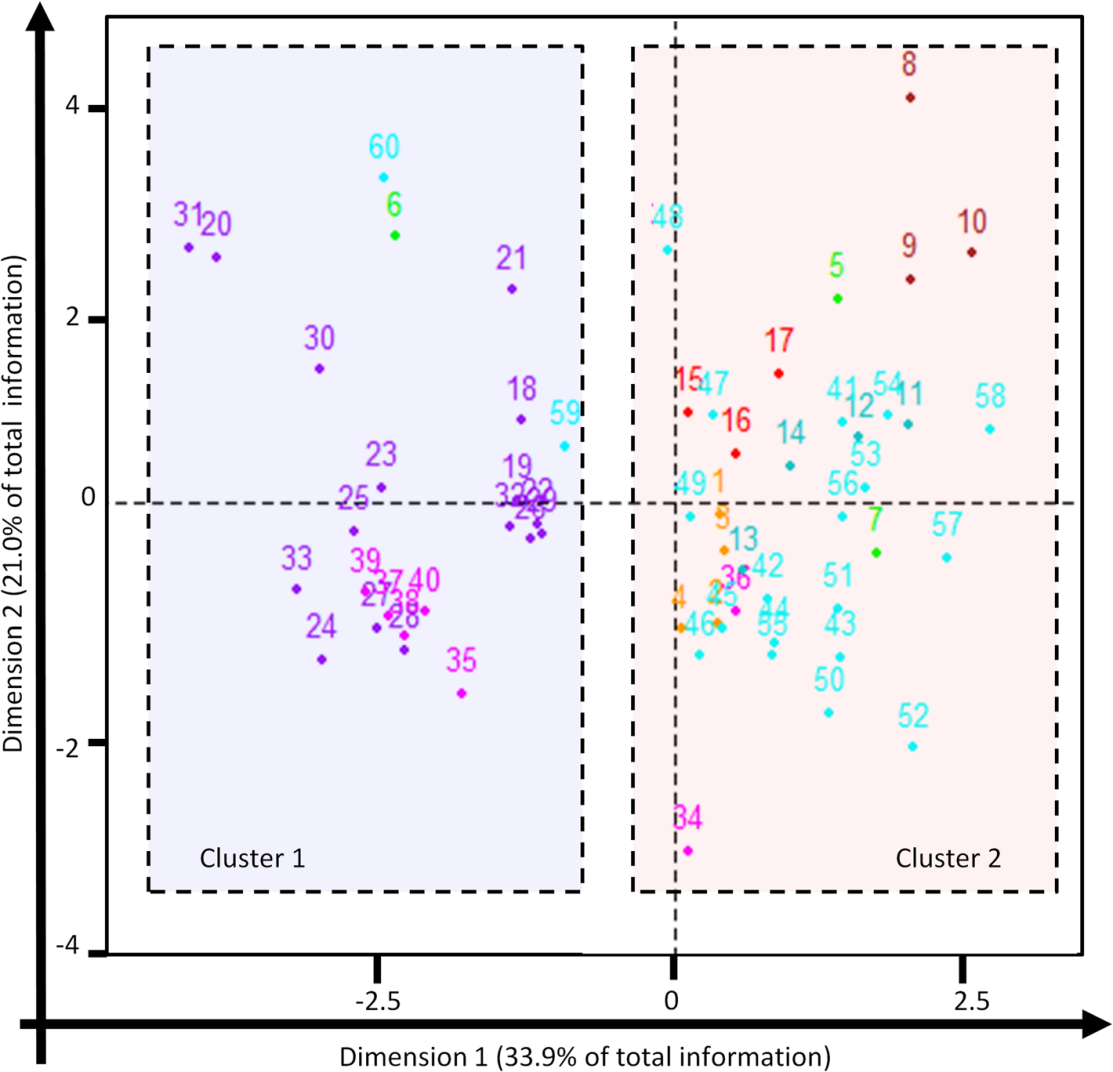
Results for the principal component analysis and hierarchical clustering. They were realized with the variables: HT quantities and proportions in the studied genomes and their partners. The individuals represented are the 60 bacteria where HT were identified, the *colors of points* and numbers indicate their habitat. Two clusters were defined (*transparent color squares*) according the bacterial habitats (Chi2 test = 2.4*10-7): 91.3% of bacteria from vertebrates are gathered in the cluster 1 (*blue square*) and 92.0% of environmental bacteria belong to the cluster 2 (*red square*). Axis 1 contains 33.9% of the information and mainly represents the transfer partners (Variable represented: *Cyanobacteria*, *Firmicutes*, *Fusobacteria* and *Alphaproteobacteria* (>40%), *Actinobacteria*, *Acidobacteria* and *Epsilonbacteria* (>20%)). The axis 2 contains 21.0% of total information and represents mainly the HT quantities and proportions (Variables represented: Sequences quantities and proportions (>70%), *Actinobacteria* and *Gammaproteobacteria* (>15%)). However, 46.1% of the information is missing, concerning mainly the *Gammaproteobacteria* and *Acidobacteria*

Most of the horizontal exchanges were realized with bacteria (94.9%) and very few with *Archaea* (2.4%), *Eukaryota* (2.5%) and viruses (0.2%). Bacteria from amoeba and ubiquitous bacteria showed a significantly higher quantity of HT instances realized with eukaryotes, compared to bacteria from other habitats (9.8 and 4.2%, respectively) (Kruskal test : *p*-value = 3.3*10-3). The proportion of exchanges with *Archaea* was significantly higher in bacteria from water-soils and from water (3.4 and 3.4%, respectively) compared to bacteria from other habitats (Kruskal test: *p*-value = 3.1*10-2). The most common bacterial partners identified were *Proteobacteria* (42%), *Firmicutes* (23%) and *Cyanobacteria* or *Actinobacteria* (6%) (Fig. 4). For several transfer partners, we identified significant differences among the 64 bacteria studied, according to their habitat: bacteria from intracellular and extracellular vertebrates were both characterized by their preference for the *Firmicutes* (21.4 and 41.1%, respectively) as transfer partners (Kruskal test : *p*-value = 3.6*10-3), and their significantly lower proportion of transfers with *Actinobacteria* (ANOVA test : *p*-value = 3.8*10-2), compared to bacteria from other habitats. Extracellular bacteria from vertebrates also presented a significantly higher proportion of transfers with *Fusobacteria* (3.0%) compared to bacteria from other habitats (Kruskal test: *p*-value = 1.6*10-2), whereas the bacteria living in soil, or soil and water, exchanged significantly more with *Acidobacteria* (4.1 and 3.9% respectively) (Kruskal test: *p*-value = 5.3*10-4) (Fig. 4). The Principal components analysis (PCA) of data recovered for HT (HT proportions and partners) showed a relationship between bacterial habitats, the quantity of HT events, and its proportion in the genome and the partner transfers (Correlation test: *p*-value = 2.4*10-7). Hierarchical clustering analysis allowed the identification of two major clusters: the environmental bacteria (soils, water, soils-water and Ubiquitous bacteria) and bacteria from amoeba were in a first cluster, and the intracellular and extracellular bacteria of vertebrates were in the second cluster (Fig. 5). A clustering according to phylogenetic relationships among species can also be identified, but was less significant (Correlation test: *p*-value = 3.5*10-4) than clustering by habitat.

### Horizontally transferred functions and phenotype

When analyzing the functions of the transferred sequences, we found that the general function distribution in the HT for the different habitats was not similar to that of the whole genomes which suggests that HT was not due to chance. Genes involved in cell processes and signaling (33 to 50%) seemed to be significantly more subject to HT, whereas genes dedicated to information storage (12 to 17%) were less subject to HT (t-test between whole genomes and transferred genes : *p*-value = 4.6*10-2 and 8.3*10-4) (Fig. 3). Moreover, there were significant differences among the habitats. Biological functions of transferred sequences were biased to three categories according to bacterial habitat: the signal transduction mechanism function in ubiquitous bacteria and in bacteria from soils (20.2 and 17.9%, respectively; ANOVA test: *p*-value = 2.3*10-4) the transport and metabolism of amino acid in bacteria from amoeba and lipids in ubiquitous bacteria (16%, Kruskal-Wallis test and correlation test: *p*-value = 7.5*10-2 and 2.1*10-4; 10.5%, ANOVA test: *p*-value = 6.9*10-3, respectively) and the defense mechanism in bacteria from extracellular vertebrates (4.7%, Kruskal-Wallis: *p*-value = 3.5*10-2).

**Fig. 6:**
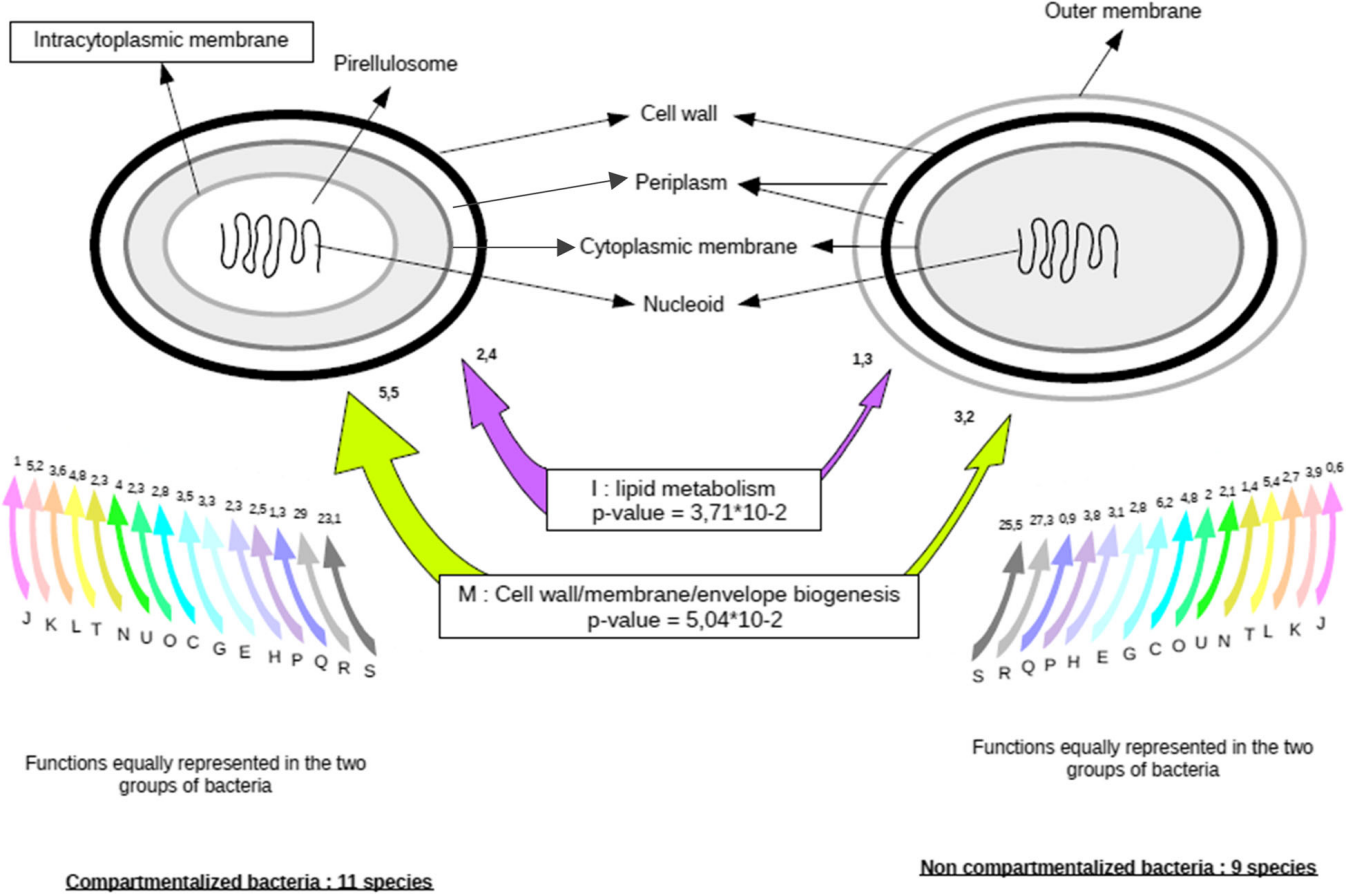
Overrepresented functions among genes transferred in compartmentalized bacteria from water. Compartmentalized bacteria are represented on the *left* of figure, non compartmentalized bacteria are represented on the *right. Color arrows* on the sides indicate the functions with similar proportions in the two groups of bacteria, these proportions are indicated close to the *arrows. Colored arrows*, in the center, show the functions that are over-represented in compartmentalized bacteria, with their complete name and the *p*-value of comparison test

To test the impact of compartmentalization on HT, we compared the 11 compartmentalized with 9 non-compartmentalized bacteria in water, where the sample size allowed us to obtain statistically significant results (Fig. 3). HT proportions and preferences for partners were identical between the two groups of bacteria. However, we detected two functions that were overrepresented among the HT events of compartmentalized bacteria, compared to non-compartmentalized, including cell wall/membrane/envelope biogenesis (5.5% of transferred genes, ANOVA test: *p*-value = 5.05*10-2) and lipid biosynthesis (2.4% of transferred genes, ANOVA test : *p*-value = 3.7*10-2) (Fig. 6). Genes encoding for these two functions were found to be horizontally transferred in compartmentalized bacteria of the different habitats, including 25 genes implicated in cell wall/membrane/envelope biogenesis (of which 12 are present in at least, one compartmentalized bacteria from each phylum) and 13 genes implicated in lipid biosynthesis (of which 3 were present in at least one compartmentalized bacteria from each phylum). Moreover, five of the genes implicated in the two functions of interest were found to be horizontally transferred in at least one compartmentalized bacteria from each habitat. These genes encoded for two different glycosyl transferases (Gene ID: 1791906 and 1796578), a carboxy-terminal processing protease (Gene ID: 1796308), an ACP reductase (Gene ID: 1793322), and a Resistance-Nodulation-Cell Division (RND) efflux membrane fusion protein (Gene ID: 1796098).

## Discussion

The comparative analysis of 33 genomes from PVC species from four different phyla showed the influence of the living environment and compartmentalization on the genome composition of PVC bacteria. The common genes were genes encoding for transcription, signal transduction mechanisms, energy production and membrane biogenesis. Conversely, shared and specific genes encode for different functions in relation to the lifestyle of the corresponding species. Evidence for a random horizontal transfer of DNA sequences has been given using a phylogenomic approach. Genes implicated in cell wall/membrane/envelope biogenesis, and those involved in lipid metabolism, were found to be over-represented among the transferred genes of compartmentalized bacteria from different habitats, according to a convergent evolutionary selection.

Our findings replicate observations from previous studies which demonstrated the role played by shared genomes in environmental adaptation [68]. Nevertheless, our approach, by examining as many as 8 different habitat conditions, offers a large advantage over other genomic studies, and increases the reliability of our results. The low proportion of specific genes that have been detected in bacteria from insects, soils and soil-water milieu is rather due to the higher number and more distant phylogenetic relationships among the species studied [69, 70] compared to the other habitats. Intracellular bacteria from vertebrates showed a low proportion (1.9%) of horizontally transferred sequences compared to the other bacteria. This result is probably related to the physical isolation of intracellular bacteria, which prevents opportunities for HT [71, 72]. This agrees with previous studies showing that the predominant evolutionary process in intracellular bacteria is genome reduction, leading to smaller genome sizes [73, 74]. Intracellular bacteria in amoeba with 4.8% of HT are the exception [75, 76], since amoeba can phagocyte several bacteria at once, giving a particular field for potential genetic exchange and a training ground for the emergence of parasitism [75].

Likewise, results obtained for partners of transfers analysis were in agreement with previous results concerning transfers between PVC bacteria and *Proteobacteria* [43] or *Spirochaetes* and *Firmicutes* [12, 13]. Indeed, HT occurred preferentially between bacteria from the same habitats, as had already been assumed. *Firmicutes* are one of the two major phyla present in the gut microbiome [77], and this is the main partner of our bacteria from vertebrates. In the same way, *Acidobacteria* are mainly detected in soil [78] and they are overrepresented as HT partners of bacteria from soils, compared to bacteria from other habitats. The tendency of bacteria from Amoeba to exchange more with Eukaryotes, especially plants, is probably due to their ancestral habitats. Indeed, ancestral *Chlamydiae* are known to have lived in and exchanged genes with the *Archaeplastides* [79, 80]. Thus, we can support the hypothesis that part of the HT detected was acquired by the interaction between the ancestors of the *Chlamydiae* and the plants, followed by the loss in the majority of bacteria. It is worth noting that like previous studies for HT detections, it is difficult to distinguish between ancient and recent HT events; yet HT partners are the witnesses of modern and ancestral habitats of the bacteria studied, and our HT analysis helps infer the ancestral habitat of these bacteria.

Beyond the complexity hypothesis that claims that genes involved in transcription and translation are less prone to transfer than metabolic genes, our findings showed that horizontal transfers can affect any function. Thus, HT do not only concern genes encoding for metabolic mechanisms and other functions that enhance pathogenicity, like genes for virulence and antimicrobial activity [1, 15, 76]; genes involved in transcription and translation, in cell surface and DNA binding, and genes essential for defense can likely be transferred as well [46, 81–83]. Positive selection might be contributing to the over-representation of some functions in the category of transferable genes [84, 85]. Indeed, horizontally acquired genes that have a useful function are maintained as it follows a strategy of colonization and adaptation to the environment. Our findings confirm previous results showing that HT particularly affects the genes involved in lipid metabolism, signal transduction and membrane transport in PVC bacteria, and genes specific to outer membrane (such as O-antigen polymerase and outer membrane efflux protein) in some *Planctomycetes* [43, 86, 87]. Since, the intracytoplasmic membrane of compartmentalized bacteria is a lipid bilayer, we can assume that the over-representation of the two functions in the genes transferred could be related to the cell plan of the bacteria. These genes may be essential for the maintenance of the supplementary intracytoplasmic membrane. Knowing that the quantity of HT events was found to be similar between compartmentalized and non compartmentalized bacteria, these results revealed the possible impact of the cell plan on the transfers’ positive selection. This selection that seems to be dependent on the function, and induces the recurrent maintenance of some transferred genes involved in the formation of compartments in bacteria from different habitats. It is noteworthy that genes implicated in lipid metabolism and membrane biosynthesis were not over-represented in the non-transferred part of the genome of compartmentalized bacteria, compared to the other bacteria; therefore, the selection seems to concern only the transferred genes.

One limitation of our comparative genomic approach is that the number of genomes studied leads to a small sample size in each environmental category, which hinders the realization of the statistical tests for certain categories. Moreover, our dataset was comprised of seven phyla, with only few representatives of soil bacteria, four for bacteria living in amoeba, and three extracellular bacteria from insects or ubiquitous, while some environmental categories contain only bacteria from just one phylum. Although the sample size was minimal, the results obtained were statistically usable and showed significant differences among phylogenetically close bacteria in relation with their habitat. Given the increased number of sequenced genomes, it will be interesting to characterize HT events in compartmentalized bacteria for diverse phyla, in order to elucidate the role of physical barriers in horizontal transfers.

## Conclusions

The genomic study of bacteria allowed to better understand the influence of the different constraints acting on genomes evolution in bacteria, especially the impact of the habitat and the special cell plan, in PVC super-phylum. The habitat influences the flux of horizontal transfer and determines the partners for genetic exchanges. The presence of an intracytoplasmic membrane in some PVC bacteria doesn’t seem to limit the HT but rather, induces a selection of transferred genes, according to their functions.

## Additional files

Additional file 1: Genomic features and habitats of bacteria studied. For each of the bacteria studied, we present the genomes identifiers, genomic features (proteins quantity, GC percent), the habitat (environment and lifestyle) and the cell plan. (CSV 6 kb)
Additional file 2: Proteins used for phylogenetic reconstruction. For each of the 768 sequences used for alignments and phylogenies we retrieved the accession number (column 1), the name of marker (column 2) and the species (column 3). (CSV 37 kb)
Additional file 3: Phylogeny of PVC bacteria and phylogeny of *Bacteroidetes-Chlorobi-Spirochaetes*. The phylogenies of studied bacteria were realized with Maximum likelihood method, within Mega 5 software, the bootstraps values are indicated at each node. The trees of PVC bacteria and *Bacteroidetes-Spirochaetes-Chlorobi* were rooted with *Bacteroides xylanosolvens* (*Spirochaetes*) and with *Bastopirellula marina* (*Planctomycetes*), respectively (the outgroup was removed for more clear representation). (TIF 2084 kb)
Additional file 4: The two phylogenetic patterns used for HT detection. The presence of one of these two patterns in a species tree allows us to identify an HT, these patterns are illustrated with examples specific to our data. (TIF 898 kb)
Additional file 5: Clustering of species according orthologous groups distribution. Clustering of studied species, according the pattern of presence/absence of genes in orthologous groups. The habitats of species are indicated by colors (red: ubiquitous, cyan: water, green: insects, yellow: amoeba, pink: intracellular vertebrates, purple: extracellular vertebrates, blue: water and soils, brown: soils) and phylum of species are in brackets. (TIF 1303 kb)
Additional file 6: HT distribution in habitats. The different boxplots describe the distribution of the numbers (a) and the proportions (b) of HT in each habitat. (TIF 878 kb)
Additional file 7: HT count according to genome size in studied bacteria. The trend curve (linear), its function (y) and the determination of coefficient (R^2^) are indicated on the plot. (TIF 606 kb)
