# Supplementary figures and images for: Identification of constraints influencing the bacterial genomes evolution in the PVC super-phylum
Source: BMC Evol Biol. 2017 Mar 9;17:75. doi: 10.1186/s12862-017-0921-3 (PMC5343374; doi:10.1186/s12862-017-0921-3)

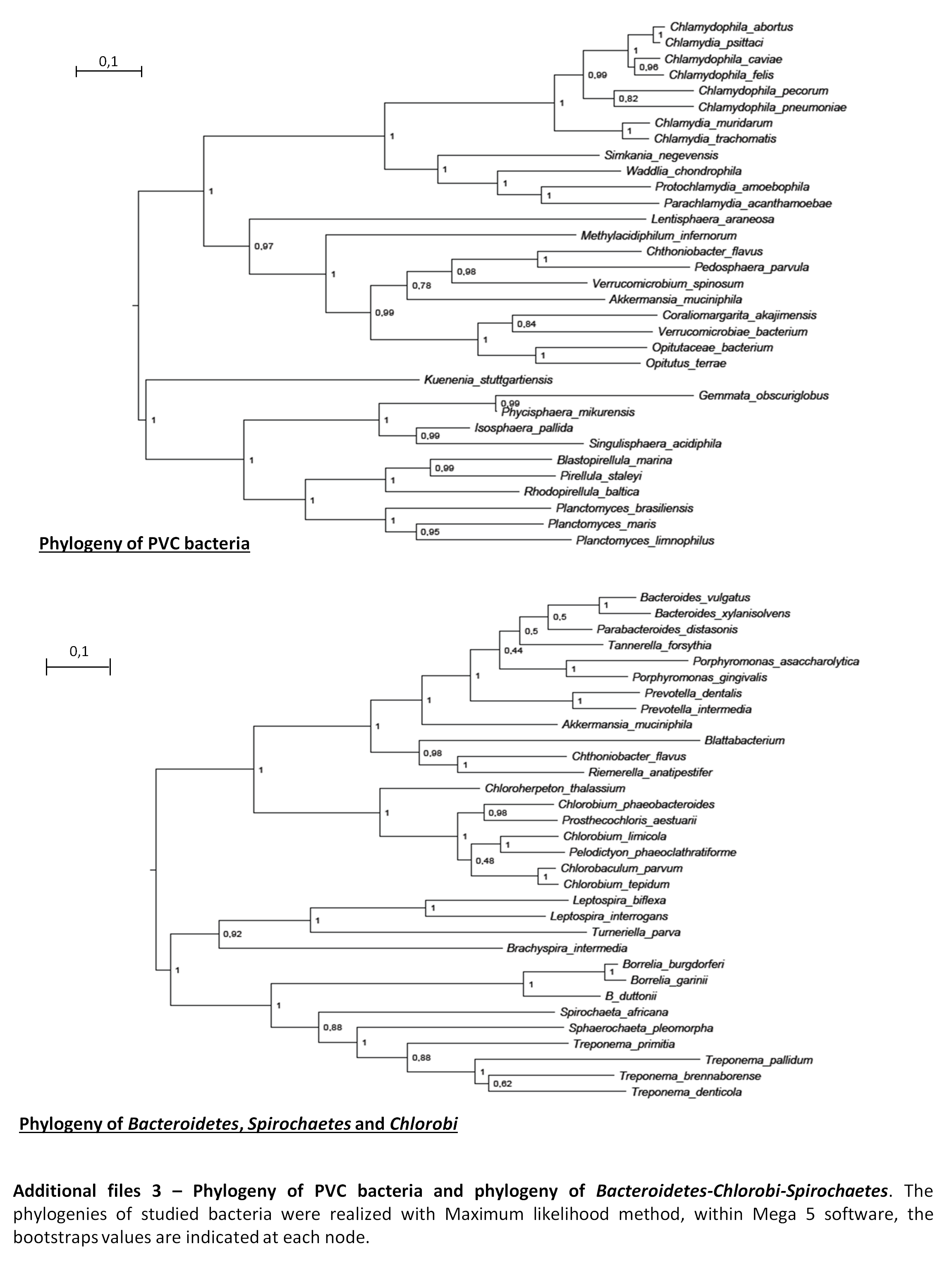

Supplement: Additional file 3: — Phylogeny of PVC bacteria and phylogeny of Bacteroidetes-Chlorobi-Spirochaetes. The phylogenies of studied bacteria were realized with Maximum likelihood method, within Mega 5 software, the bootstraps values are indicated at each node. The trees of PVC bacteria and Bacteroidetes-Spirochaetes-Chlorobi were rooted with Bacteroides xylanosolvens (Spirochaetes) and with Bastopirellula marina (Planctomycetes), respectively (the outgroup was removed for more clear representation). (TIF 2084 kb) [file 12862_2017_921_MOESM3_ESM.tif]

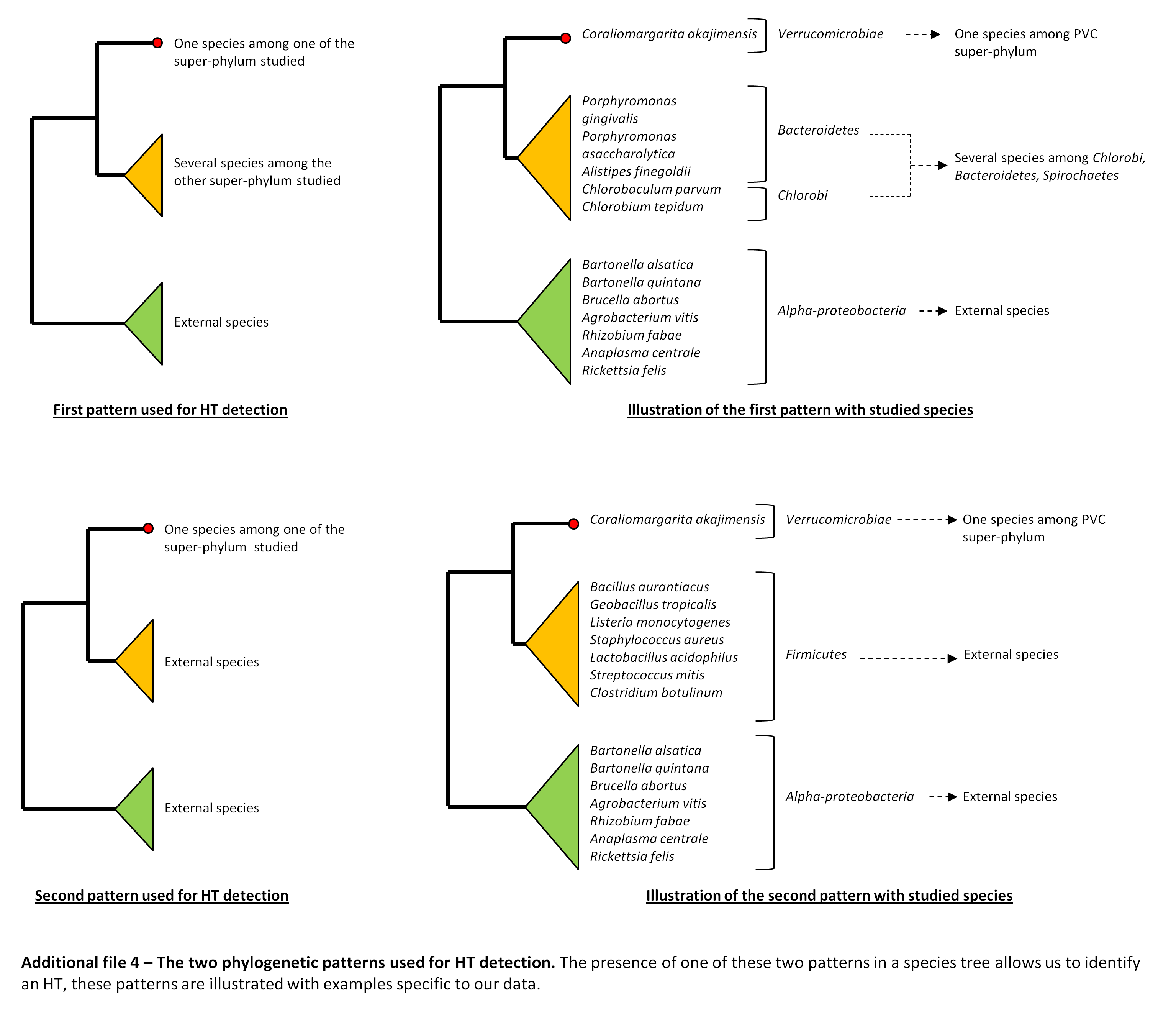

Supplement: Additional file 4: — The two phylogenetic patterns used for HT detection. The presence of one of these two patterns in a species tree allows us to identify an HT, these patterns are illustrated with examples specific to our data. (TIF 898 kb) [file 12862_2017_921_MOESM4_ESM.tif]

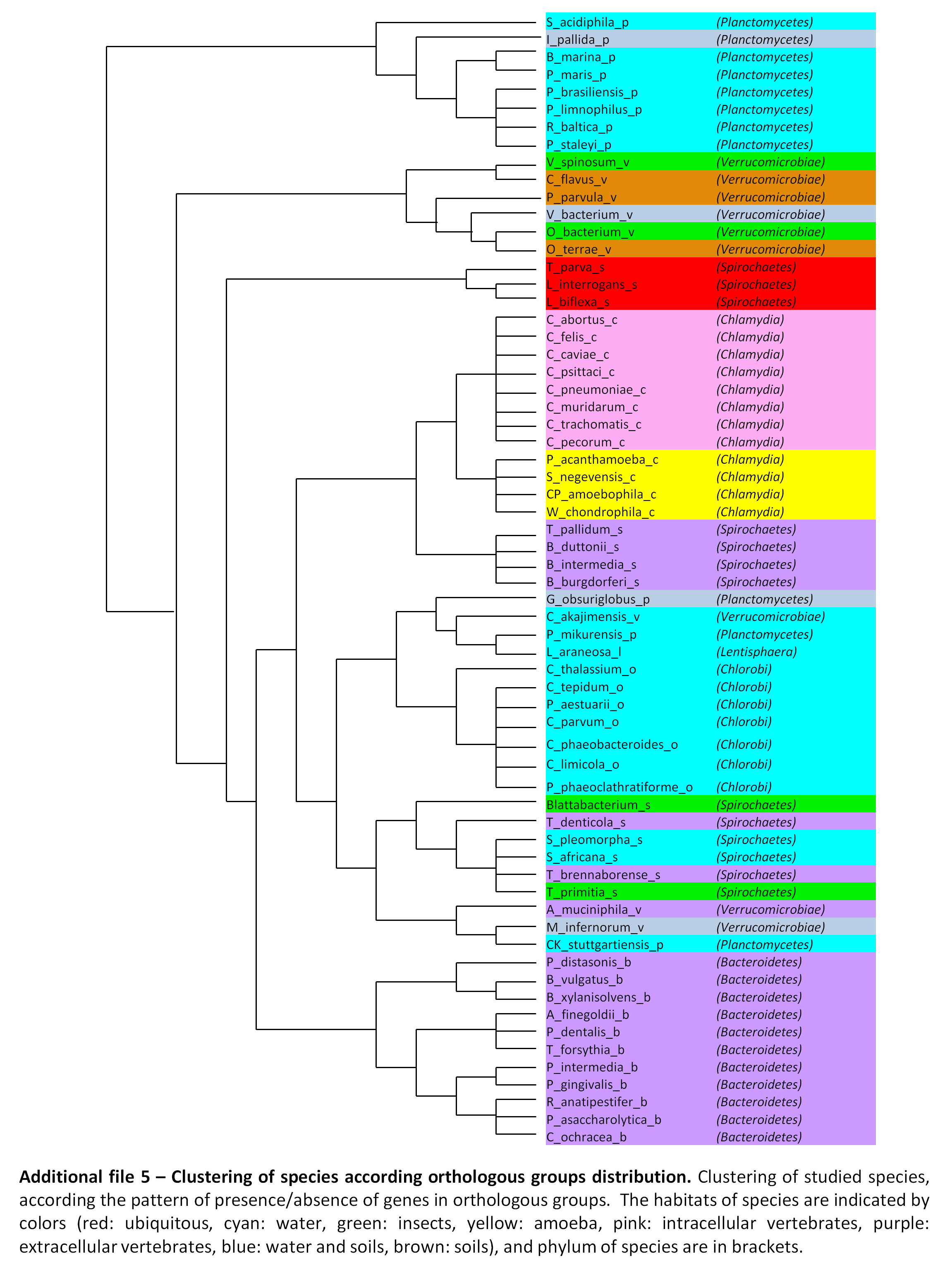

Supplement: Additional file 5: — Clustering of species according orthologous groups distribution. Clustering of studied species, according the pattern of presence/absence of genes in orthologous groups. The habitats of species are indicated by colors (red: ubiquitous, cyan: water, green: insects, yellow: amoeba, pink: intracellular vertebrates, purple: extracellular vertebrates, blue: water and soils, brown: soils) and phylum of species are in brackets. (TIF 1303 kb) [file 12862_2017_921_MOESM5_ESM.tif]

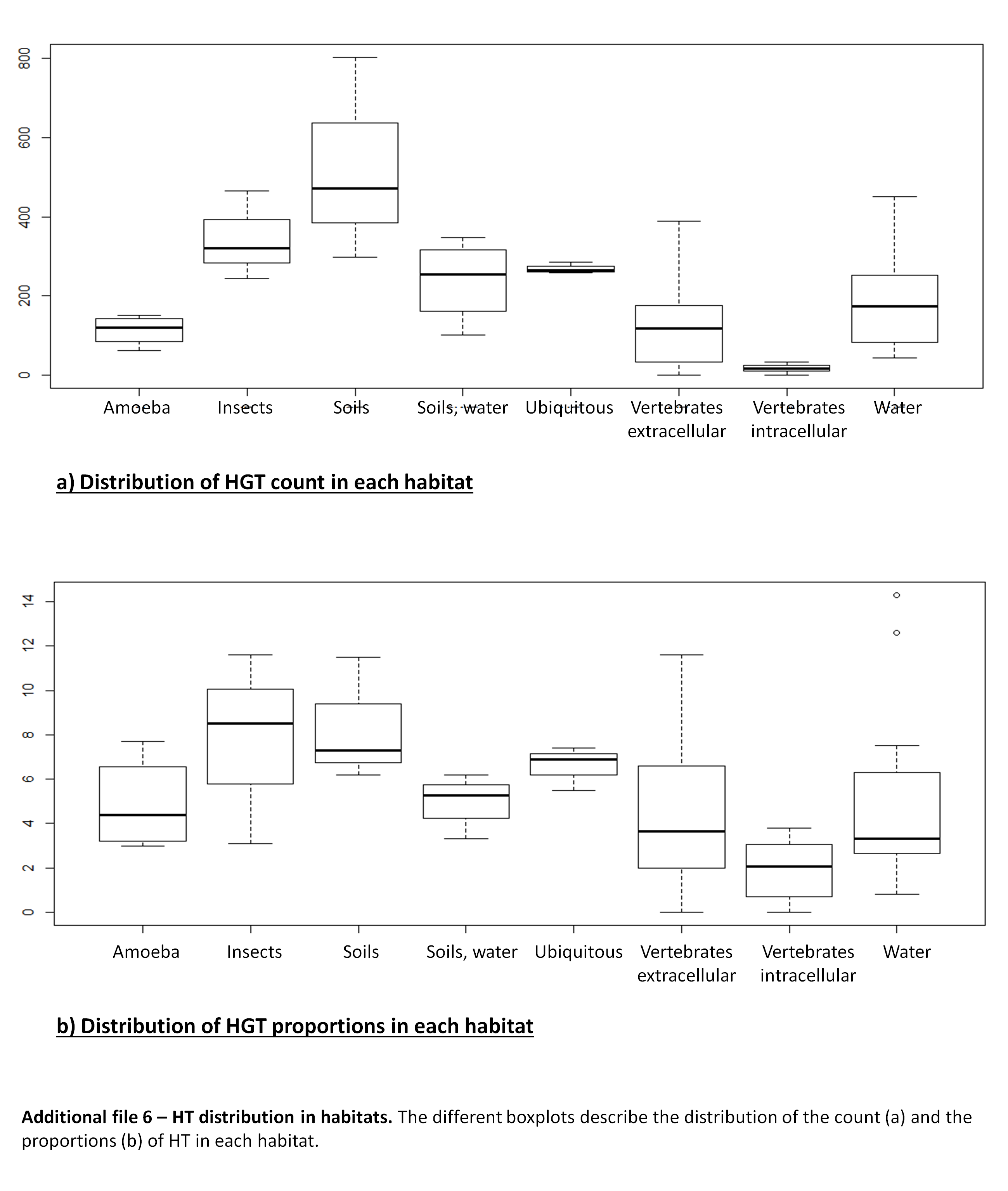

Supplement: Additional file 6: — HT distribution in habitats. The different boxplots describe the distribution of the numbers (a) and the proportions (b) of HT in each habitat. (TIF 878 kb) [file 12862_2017_921_MOESM6_ESM.tif]

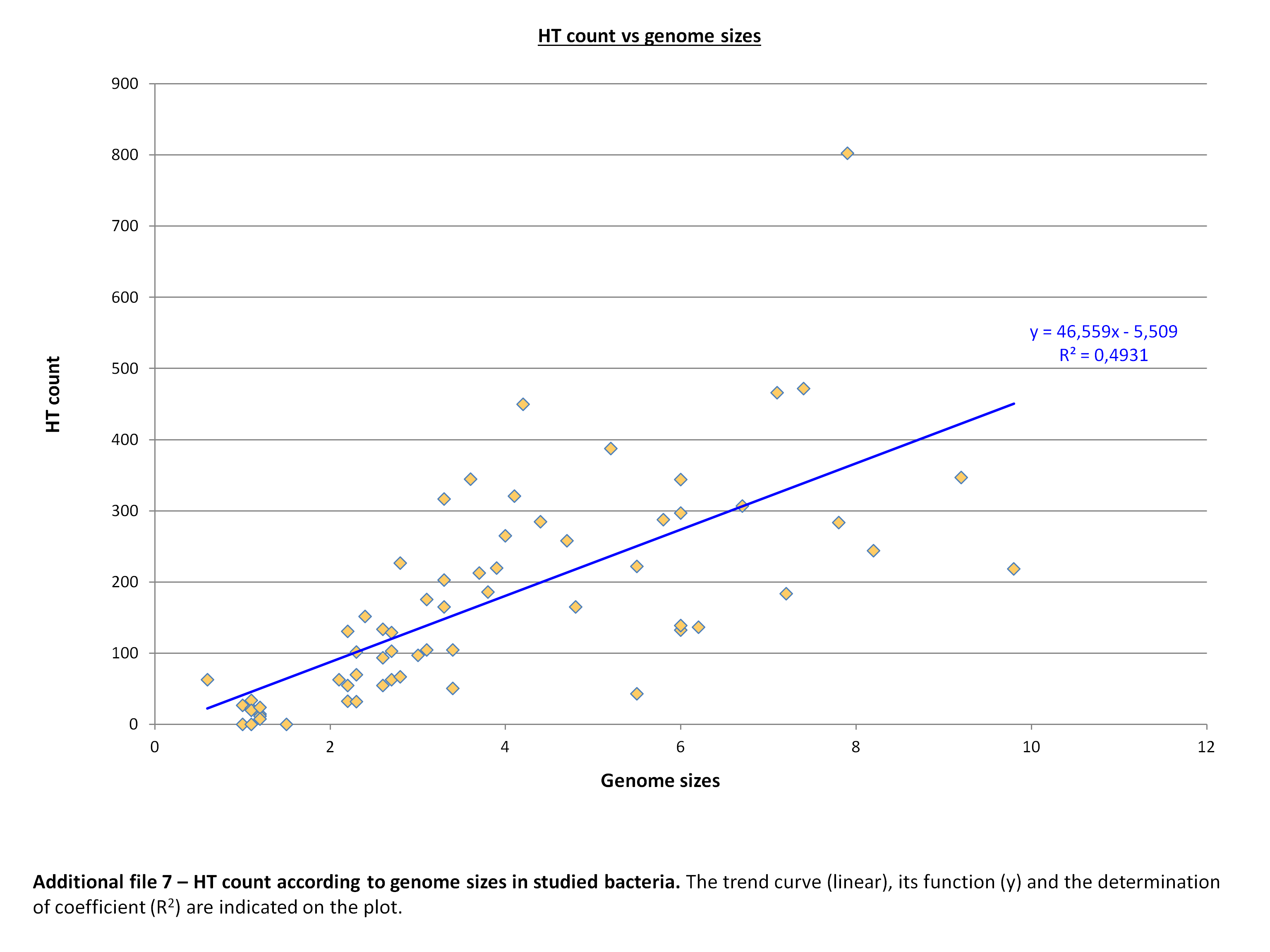

Supplement: Additional file 7: — HT count according to genome size in studied bacteria. The trend curve (linear), its function (y) and the determination of coefficient (R2) are indicated on the plot. (TIF 606 kb) [file 12862_2017_921_MOESM7_ESM.tif]
